# Bioinspired Cellulase-Mimetic Solid Acid Catalysts for Cellulose Hydrolysis

**DOI:** 10.3389/fbioe.2021.770027

**Published:** 2021-11-18

**Authors:** Guangxu Yang, Xiaolin Luo, Li Shuai

**Affiliations:** College of Materials Engineering, Fujian Agriculture and Forestry University, Fuzhou, China

**Keywords:** glucose, cellulase, solid acid, cellulase-mimetic catalysts, cellulose hydrolysis

## Abstract

Glucose produced by catalytic hydrolysis of cellulose is an important platform molecule for producing a variety of potential biobased fuels and chemicals. Catalysts such as mineral acids and enzymes have been intensively studied for cellulose hydrolysis. However, mineral acids show serious limitations concerning equipment corrosion, wastewater treatment and recyclability while enzymes have the issues such as high cost and thermal stability. Alternatively, solid acid catalysts are receiving increasing attention due to their high potential to overcome the limitations caused by conventional mineral acid catalysts but the slow mass transfer between the solid acid catalysts and cellulose as well as the absence of ideal binding sites on the surface of the solid acid catalysts are the key barriers to efficient cellulose hydrolysis. To bridge the gap, bio-inspired or bio-mimetic solid acid catalysts bearing both catalytic and binding sites are considered futuristic materials that possess added advantages over conventional solid catalysts, given their better substrate adsorption, high-temperature stability and easy recyclability. In this review, cellulase-mimetic solid acid catalysts featuring intrinsic structural characteristics such as binding and catalytic domains of cellulase are reviewed. The mechanism of cellulase-catalyzed cellulose hydrolysis, design of cellulase-mimetic catalysts, and the issues related to these cellulase-mimetic catalysts are critically discussed. Some potential research directions for designing more efficient catalysts for cellulose hydrolysis are proposed. We expect that this review can provide insights into the design and preparation of efficient bioinspired cellulase-mimetic catalysts for cellulose hydrolysis.

## Introduction

Glucose, resulting from hydrolysis of cellulose, is an important biomass-derived platform molecule for producing a variety of value-added fuels and chemicals (Yabushita et al., 2014). Many efforts are ongoing to reduce glucose production cost for economic biorefineries. To a large extent, the efficiency of catalysts for cellulose hydrolysis affects the economy of glucose production. A good catalyst should selectively and cost-effectively convert cellulose to glucose in high concentrations with limited glucose degradation.

Mineral acids and enzymes (such as cellulase) have been widely used as catalysts for cellulose hydrolysis. Despite the low cost of mineral acids, mineral acid-catalyzed cellulose hydrolysis has issues such as glucose degradation, equipment corrosion, and wastewater treatment (Palkovits et al., 2010; Orozco et al., 2011; Qiao et al., 2018; Zhou et al., 2021). In contrast, cellulase can selectively catalyze cellulose hydrolysis at mild conditions but the cost of cellulase loadings required for efficient conversion of cellulosic materials to glucose accounts for a large portion of the whole processing cost. Moreover, cellulase has the highest catalytic activity only under an optimum condition; a varied condition (e.g. a higher or lower temperature or pH value) inhibits cellulase activity and even denatures cellulase; therefore, the rate of enzymatic hydrolysis cannot be accelerated through an increase in the reaction temperature (Berlin et al., 2007; dos Santos et al., 2018; Huang, 1975). In consideration of recyclability and stability, solid acid catalysts have been widely explored in recent years while the efficiency of cellulose hydrolysis catalyzed by these catalysts is highly restricted by the limited interactions between the catalysts and cellulose (Hara, 2010; Shimizu et al., 2011; Huang et al., 2013; Hu et al., 2015; Wang et al., 2015) To overcome this limitation, novel cellulase-mimetic catalysts with both cellulose-binding and catalytic sites were developed in the last decade (Shuai et al., 2012). The catalytic sites consisting of acid-base pairs can stabilize the intermediate product (i.e., an oxocarbenium ion) during the cleavage of the glycosidic bond, thereby reducing the energy barrier of cellulose hydrolysis; the binding group on the surface of a cellulase-mimetic catalyst can associate the catalyst with cellulosic materials, promoting the mass transfer rate between the catalyst and cellulose in such a heterogeneous reaction.

In this review, we summarize the recent studies regarding cellulase-mimetic catalysts. In particular, the mechanism of cellulase-catalyzed cellulose hydrolysis and the structural characteristics of cellulase-mimetic catalysts such as binding and catalytic sites are critically reviewed. In the end, we discuss the issues of the current catalyst design and propose some potential directions for synthesizing more efficient cellulase-mimetic catalysts.

## Cellulase-Catalyzed Cellulose Hydrolysis

**FIGURE 1:**
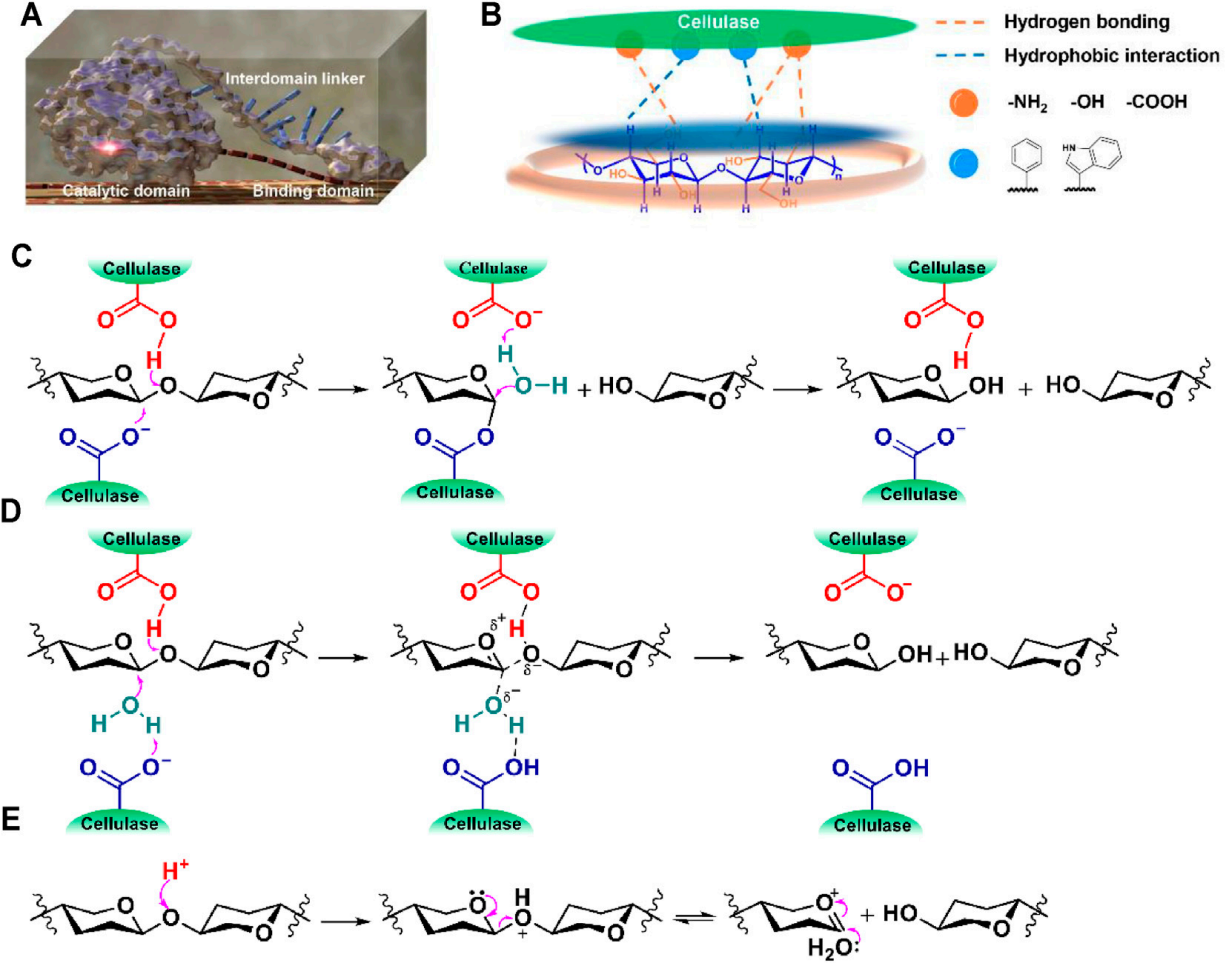
**(A)** A modeling image of cellulase, **(B)** cellulase-cellulose binding *via* hydrogen bonding (orange) and hydrophobic interactions (blue), catalytic hydrolysis of cellulose by cellulase in **(C)** a retaining or **(D)** an inverting mechanism, and **(E)** a proposed mechanism of mineral acid-catalyzed cellulose hydrolysis (substituted groups are omitted for better visibility).

Structurally, cellulase comprises at least three separate structural elements of different functions, i.e., a catalytic domain (CD), a cellulose-binding domain (CBD), and an interdomain linker (Figure 1A; Rabinovich et al., 2002). The CBD is responsible for associating cellulase with cellulose while the CD catalyzes the cleavage of glycosidic bonds of a cellulose chain. During the enzymatic hydrolysis of cellulose, cellulase is adsorbed onto the bulk solid cellulosic materials through hydrophobic interactions (e.g., CH–π interaction) between the CBD containing the aromatic and alkyl residues of amino acids and the axial face of cellulose as well as hydrogen bonding interactions between the polar groups of amino acids and cellulose hydroxyls (Figure 1B; Asensio et al., 2012; Georgelis et al., 2012), then the cleavage of the glycosidic bond of the captured cellulose chain is catalyzed by a catalytic site in a retaining or inverting mechanism (Figures 1C,D; Badieyan et al., 2012; Zechel et al., 2000). In both of the proposed hydrolysis mechanisms, a carboxylic acid (COOH)-carboxylate (COO^−^) pair (an acid-base pair) works synergistically to cleave the glycosidic bond (Figures 1C,D). The nucleophilic attack of COO^−^ to the anomeric carbon of cellulose stabilizes the high-energy free oxocarbenium ions that is usually formed in an acid-catalyzed hydrolysis process (Figure 1E; Brown et al., 1979; Rinaldi et al., 2010), thereby reducing the energy barrier for cellulose hydrolysis. During the enzymatic hydrolysis of cellulose, the occurrence of such an enzymatic reaction highly depends on the perfect conformation of the acid-base pair. When the glycosidic bond of a cellulose chain is perfectly fit into the acid-base pairing site, the energy barrier for cellulose hydrolysis is substantially lowered. Such perfect fitting is assisted by the CBD which associates cellulase with cellulose chains *via* specific adsorption. The adsorption of cellulase to cellulose increases the mass transfer rate between them, thereby further increasing the enzymatic hydrolysis efficiency.

A temperature or pH change would substantially affect the structural conformations of the catalytic and binding sites and thereby the catalytic activity of cellulase. The hydrolysis rate of cellulose is thus strictly limited by the optimum temperature for cellulase and cannot be improved *via* changing the reaction temperature. In contrast, a temperature increase can enhance the rate of acid-catalyzed hydrolysis without changing the structure of chemically synthesized solid acid catalysts. Therefore, solid acid catalysts are receiving increasing attention due to its high potential to overcome the limitation of cellulase.

## Cellulase-Mimetic Catalysts

As conventional solid acids do not have specific sites for binding cellulose and specific acid-base pairs for catalyzing cellulose hydrolysis, the rate of cellulose hydrolysis highly relies on the mass transfer rate between the acid catalysts and cellulose as well as the acidity of the catalysts.

Particularly, the mass transfer rate is highly restrained when a solid acid is used as a catalyst. An elevated temperature could enhance the mass transfer rate but it would cause more cellulose and glucose degradation and thereby low glucose selectivity (Zuo et al., 2014). Alternatively, synthesis of cellulase-mimetic solid acid containing both catalytic and cellulose-binding sites (groups) is a viable way to improve the cellulose hydrolysis efficiency.

### Mimetic Design of Catalytic Sites

**FIGURE 2:**
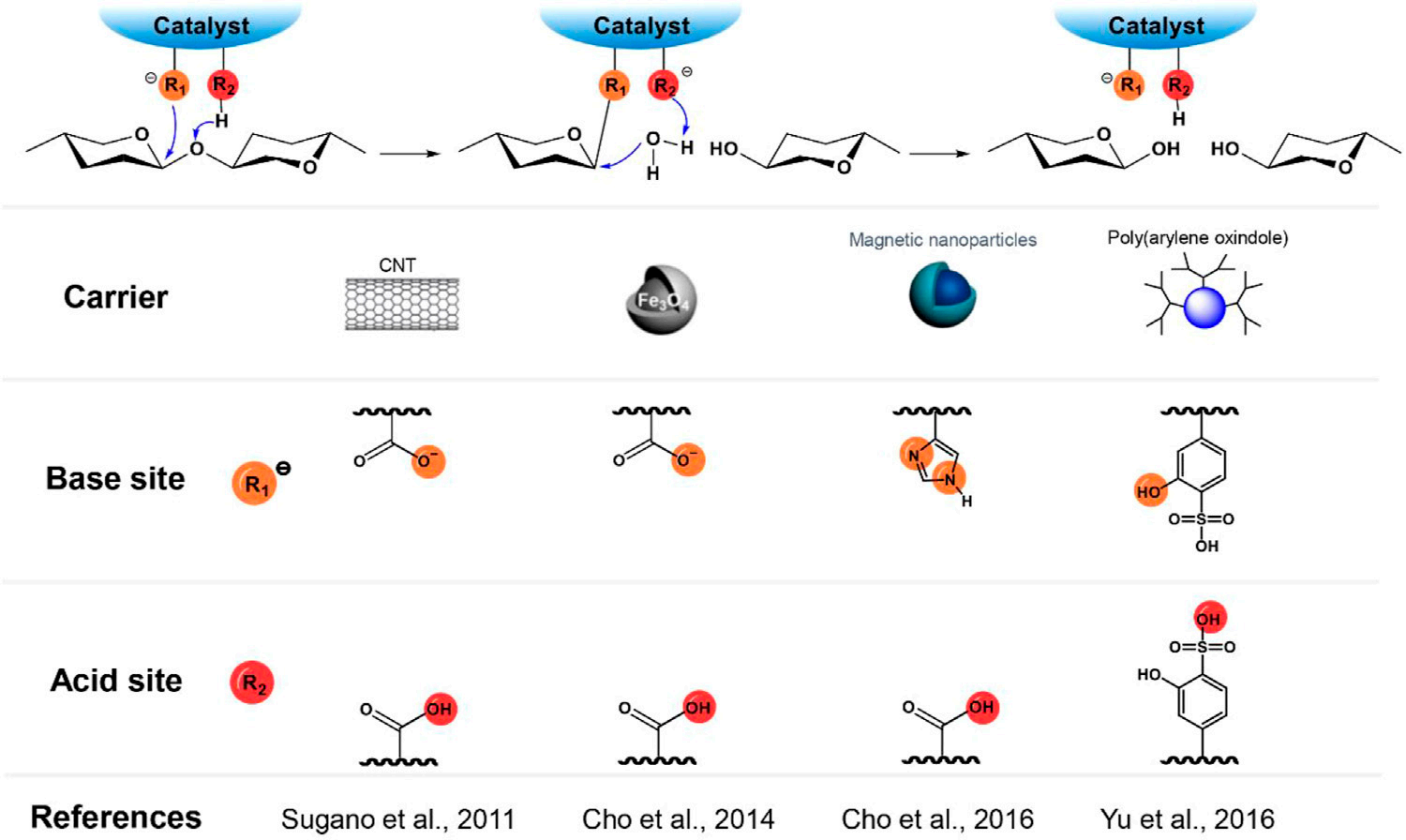
A summary of acid-base pairs as catalytic sites of cellulose-mimetic solid acid catalysts and corresponding mechanism for the catalytic cleavage of glycosidic bonds in cellulose chain.

The mechanism of cellulase-catalyzed cellulose hydrolysis indicates the important role of the COOH-COO^−^ pair in the cleavage of the glycosidic bonds. Incorporation of COOH-COO^−^ (Cho et al., 2015; Sugano et al., 2011) or similar acid-base pairs (e.g. COOH-NH_2_) (Chen et al., 2019; Cho et al., 2016) onto catalyst supporting materials (e.g. carbon nanotubes (CNT) (Sugano et al., 2011), and magnetic silica-based nanoparticles (Cho et al., 2015), and green magnetic nano-catalyst (GMN) (Cho et al., 2016) to mimic the catalytic conformation of cellulase was therefore explored extensively (Figure 2). Since the theoretical pKa value of the carboxylic group is around 3.1–4.4, the pH value of the hydrolysis reaction media is generally adjusted to around 3.0 to ensure the presence of the acid-base (or conjugated base) pair in a reaction medium for the best performance (Cho et al., 2015; Sugano et al., 2011). In contrast to the conventional acid-catalytic hydrolysis processes that require high temperatures, some of these catalysts (e.g., a green magnetic nano-catalyst (GMN) with carboxyl and imidazole groups as an acid-base pair) could catalyze the cleavage of the glycosidic bonds under a very mild condition (37.5°C, pH = 5) in a similar manner to cellulase (Cho et al., 2016). However, these catalysts are far less active than cellulase because the random distribution of acid (such as -COOH and phenolic -OH) and base (such as COO^−^ and NH_2_) groups on the surface of the catalysts does not completely mimic the ideal structural conformation of the COOH-COO^−^ pair in cellulase. To understand the effect of well-arranged acid-base pairs on the hydrolysis efficiency, a comparative study of ortho-, meta-, and para-hydroxybenzoic acids (pKa values of 3.0, 4.1, and 4.6, respectively) as the catalysts for cellulose hydrolysis was conducted (Yabushita, 2016). The considerably higher catalytic activity of ortho-hydroxybenzoic acid (a turnover frequency (TOF) of 28 h^−1^) than others (TOFs of 5.7 and 2.4 h^−1^ for meta- and para-hydroxybenzoic acids, respectively) suggests the major contribution of a vicinal acid-base pair to the catalytic activity of a synthesized catalyst (Yabushita, 2016). The vicinal acid-base pair has a better conformation than others towards stabilizing the intermediate oxocarbenium ion and protonating the hydroxyl ion formed during the cleavage of the glycosidic bond. Consistently, a polymer acid catalyst, hyperbranched poly(arylene oxindole) (5-OH-SHPAO) functionalized with phenolic hydroxyls and ortho-substituted sulfonic acids as vicinal acid-base pairs demonstrated high catalytic activity for cellulose hydrolysis and achieved a cellulose conversion of 88% with a glucose yield of 56% and above 93% selectivity to all useful hydrolytic and dehydration products (such as lactic acid) at 170°C in 4 h (Figure 2; Yu et al., 2016). During the hydrolysis, the phenolic hydroxyl attacks the anomeric carbon to stabilize the oxocarbenium ion and the resulting hydroxyl ion can be readily protonated by the neighboring sulfonic acid. In contrast to the findings of Yabushita et al. where the strict requirement of a vicinal acid-base site is preferred for constructing the catalytic site (Yabushita, 2016), a biochar sulfonic acid bearing randomly distributed polyamide chains (BCSA-PA) also demonstrated higher activity for cellulose hydrolysis (26% reducing sugars (RSs) and 23% HMF yields) than BCSA without PA (22% RSs and <0.1% HMF yields) (Figure 2; Chen et al., 2019). The finding indicates that the flexible polyamide chain can easily move to attack the anomeric carbon to stabilize the oxocarbenium ion, reducing the energy barrier of the cleavage of the glycosidic bonds.

In summary, the merit of constructing of a catalytic acid-base pair is to stabilize the intermediate and enable the occurrence of the hydrolysis under mild conditions and even without the use of strong acid catalysts. However, it is very challenging to design a solid acid that can completely mimic the naturally evolved catalytic conformation of cellulase. Due to such a challenge, current cellulase-mimetic solid acid catalysts are not comparable to cellulase in terms of the catalytic activity.

### Mimetic Design of Binding Sites

The high efficiency of cellulase-catalyzed cellulose hydrolysis is due to not just the unique structural configuration of the catalytic site but also the assistance of the CBD in associating cellulase with cellulosic materials. In addition to the absence of ideal acid-base pairs, the slow mass transfer between solid acid catalysts and cellulose is another limitation to efficient hydrolysis. Incorporation of binding sites to a catalyst to enhance the mass transfer rate is therefore another important research area of cellulase-mimetic catalyst development. The catalytic performance of various cellulase-mimetic solid acid catalysts on hydrolyzing cellulose was compared in Table 1.

**TABLE 1:** The catalytic performance of various cellulase-mimetic solid acids catalysts on hydrolyzing cellulose.

Entry	Catalyst	CBD	Binding interaction	Substrates	Reaction condition	Conv. (%)	Glu. (%)	Other products (%)	Catalyst stability	Ref
1	Fe-GO-SO_3_H	-OH, -COOH	Hydrogen bonding	Cellulose	H_2_O, 75°C, 44 h	95	50	Cellobiose (4.3) 4-5unit oligomeric (40)	Kept stable after recycling five times	Verma et al. (2013)
2	PPSAs	-OH	Hydrogen bonding	Avicel	H_2_O, 120°C, 48 h	_	93	_	From 92.6% to 65.1% after four cycles	Yang et al. (2021)
3	CP-SO_3_H	-Cl	Hydrogen bonding	Avicel	H_2_O, 120°C, 10 h	_	93	_	The activity did not decline after recycling three times	Shuai et al. (2012)
4	PTA@MIL-101-NO_2_	-NO_2_	Hydrogen bonding	Avicel	H_2_O, 180°C, 3–11 h	_	16.2	_	Decreased by 4.3% after three times	Han et al. (2019)
5	PTA@MIL-101-Br	-Br	Hydrogen bonding	Avicel	H_2_O, 180°C, 3–11 h	_	10.1	_	Decreased by 3.7% after three times	Han et al. (2019)
6	PTA@MIL-101-NH_2_	-NH_2_	Hydrogen bonding	Avicel	H_2_O, 180°C, 3–11 h	_	12.8	_	Kept stable after recycling three times	Han et al. (2019)
7	PTA@MIL-101-Cl	-Cl	Hydrogen bonding	Avicel	H_2_O, 180°C, 3–11 h	_	15.0	_	Decreased by 3.9% after three times	Han et al. (2019)
8	CP-SO_3_H-1.69	-Cl	Hydrogen bonding	Avicel	H_2_O, 170°C, 10 h	100	2.1	LA (33.1)	Deactivated in two runs	Zuo et al. (2014)
9	SUCRA-SO_3_H	-Cl	Hydrogen bonding	Hardwood (rice straw) pretreated by the ionic liquid	H_2_O, 120°C, 24 h	_	12.7 (19.5)	Xylose (44.4, 57.3)	Remained around 56% after seven times	Hu et al. (2014)
10	HA–CC–SO_3_H	-Cl	Hydrogen bonding	Avicel	H_2_O, 155°C, 4 h	11.3	10.8	_	Kept stable after recycling three times	Pang et al. (2014)
11	SA-TsOH	-Cl	Hydrogen bonding	Avicel	[BMIM][Cl], 130°C, 1 h	_	_	TRS (67.6)	From 67.6 to 60.4% after eight cycles	Shen et al. (2018)
12	Solid acid	-Cl	Hydrogen bonding	Ball-milled Avicel	H_2_O, 120°C, 24 h	_	84.9	_	Lost activity after four runs	Yang et al. (2016)
13	Fe_3_O_4_/Cl-MCMB-SO_3_H	-Cl	Hydrogen bonding	Cellulose	H_2_O, 140°C, 3 h	_	_	TRS (68.6)	From 68.6 to 61.1% after six cycles	Li et al. (2020)
14	Cl-MCMB-SO_3_H	-Cl	Hydrogen bonding	Pretreated MCC	H_2_O, 130°C, 3 h	_	_	TRS (70.3)	Decreased by 9.6% after five times	Li et al. (2020)
15	CMC-SO_3_H	-Cl, -OH, -COOH	Hydrogen bonding	RSDC	[BMIM][Cl], 130°C, 4 h	_	_	TRS (73.2)	Kept stable after recycling five times	Hu et al. (2016)
16	SA-SO_3_H	-Cl	Hydrogen bonding	Ball-milled Avicel	H_2_O, 180°C, 12 h	_	5.7	LA (52.2)	Decreased by 11% after five times	Shen et al. (2017)
17	CCSA	-Cl, -OH, -COOH	Hydrogen bonding	Cellobiose	H_2_O, 120°C, 6 h	_	44.76	_	Leached about 10–25% of -Cl after recycling three times	Shen et al. (2014)
18	MCMPS-Cl-SO_3_H	-Cl, -OH	Hydrogen bonding	Pretreated MCC	[BMIM][Cl], 120°C, 6 h	_	_	TRS (84.7)	Kept stable after recycling three times	Ding et al. (2019)
19	BCSA-IL-Cu	IL groups	Hydrogen bonding	Bamboo pretreated by microwave	[BMIM][Cl]: H_2_O = 1:1, 120°C, 2 h	_	_	TRS (27.6) HMF (8.2)	Lost activity after four runs	Zhang et al. (2013)
20	Porous polymers solid acids	Boronic acids groups	Reversible chemical bonding	Avicel	H_2_O, 120°C, 48 h	_	94.6	_	_	Yang et al. (2016)

GO, graphene oxide; PPSAs, porous polymeric solid acids; CP-SO_3_H, sulfonated chloromethyl polystyrene resin; PTA, Phosphotungstic acid; MIL-101, a metal organic framework; LA, levulinic acid; SUCRA-SO_3_H, sucralose-derived solid acid catalyst; HA–CC–SO_3_H, hydrochloric acid-treated cellulose derived carbon solid acid catalyst; SA, sucralose; TsOH, p-toluenesulfonic acid; TRS, total reducing sugar, consisting of soluble glucose and oligosaccharides; MCMB, magnetic mesocarbon microbead; MCC, microcrystalline cellulose; CMC, chlorine-doped magnetic amorphous carbon; RSDC, rice straw-derived cellulose; CCSA, a chlorine functionalized carbon-based solid acids; MCMPS-Cl-SO_3_H, a magnetically cellulase-mimetic resin catalyst; BCSA, biochar sulfonic acid; HMF, 5-hydroxymethyl furfural; IL, 1-(trimethoxypropylsilane)-3-methyl imidazolium chloride.

### (A) Binding *Via* Hydrogen Bonding Interaction

**FIGURE 3:**
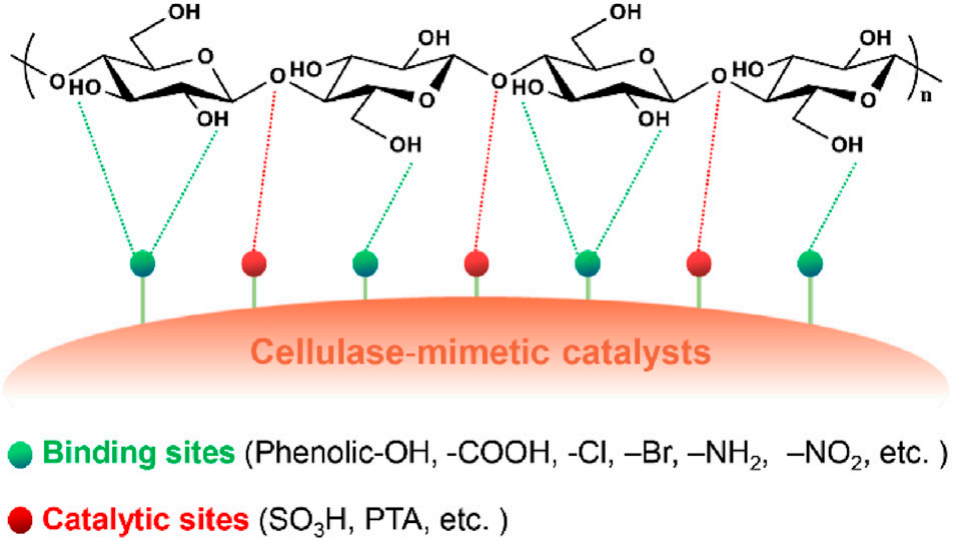
A summary of functional groups as binding sites of cellulase-mimetic solid acid catalysts and the proposed catalysis mechanism for cellulose hydrolysis.

Initially, researchers found that carbon-based solid acids prepared from incomplete carbonization of natural organic matters followed by sulfonation exhibited remarkable hydrolysis performance with water-soluble glucose and glucan yields of 4–68% (Suganuma et al., 2008; Toda et al., 2005). Further mechanistic studies revealed the contribution of the hydrogen bonding interaction between the phenolic -OH and -COOH on the catalyst surface and cellulose hydroxyls to the high catalytic activity of the carbon-based solid acids (Figure 3; Hu et al., 2015; Suganuma et al., 2008). The interaction renders enhanced mass transfer rates between the catalysts and cellulosic materials and thereby high hydrolysis efficiencies. This finding inspires studies on a variety of carbon-based catalysts prepared from different raw materials, such as mesoporous carbon (CMK-3) (Pang et al., 2010), mesoporous silicon oxide/carbon composite (Van de Vyver et al., 2010), magnetic oxide/sulfonated carbon shell composite (Zhang et al., 2013), graphene oxide (GO) (Kitano et al., 2009; Verma et al., 2013; Zhao et al., 2014; Mission et al., 2017; Zhang et al., 2017), and lignin (Hu et al., 2015; Zhu et al., 2016; Gan et al., 2017; Wang et al., 2020; Zhu et al., 2020). In these studies, GO-based catalysts demonstrate excellent performance for cellulose hydrolysis. The high surface area and abundant carboxylic and phenolic hydroxyl groups on the surface of the GO-based catalysts facilitate the formation of a considerable amount of hydrogen bonds with cellulose (Zhao et al., 2014). The apparent activation energy for cellulose hydrolysis catalyzed by a functionalized GO/iron nanoparticle hybrid material (Fe-GO-SO_3_H) was only 12 kJ mol^−1^, which is much lower than that for sulfuric acid-catalyzed hydrolysis under an optimal condition (170 kJ mol^−1^) (Verma et al., 2013). Recently, Yang et al. (Yang et al., 2021) also prepared porous polymeric solid acids (PPSAs) bearing hydroxyl and sulfonic acid groups for cellulose hydrolysis in water through the low-cost Friedel-Crafts “knitting” polymerization of hydroxyl-containing aromatic monomers followed by sulfonation. The reason for the high efficiency of the synthesized bifunctional catalysts (a glucose yield of 93% from Avicel at 120°C within 48 h) was attributed to the porous structure and the presence of the hydroxyl (cellulose-binding group) on the solid acids.

The enhanced performance resulting from the hydrogen bonding interaction between carbon-based catalysts and cellulosic materials encouraged many attempts to examine other electronegative groups such as amine, and halide as cellulose-binding groups. The apparent activation energies for the hydrolysis of cellobiose and microcrystalline cellulose (Avicel) catalyzed by a polymer-based catalyst bearing chlorine (-Cl) and -SO_3_H (CP-SO_3_H) were reduced to 78 and 83 kJ mol^−1^, respectively, which are much lower than those of sulfuric acid-catalyzed hydrolysis (133 and 170 kJ mol^−1^, respectively) (Figure 3; Shuai et al., 2012). The CP-SO_3_H catalyst adsorbed both cellobiose and glucose and meanwhile showed higher affinity to cellobiose, which is presumably because more hydrogen bonds formed between the catalyst and cellobiose due to more hydroxyl groups available in cellobiose. The catalysts bearing -NH_2_ and -OH groups showed lower activity than that bearing -Cl but higher catalytic activity than support (Amberlyst-15) without any binding and catalytic sites (Shuai et al., 2012). This comparison indicates that a more electronegative group has higher affinity to cellulosic materials. Similarly, a metal-organic framework material (MIL-101) carrier bearing various electronegative groups (X = -Br, -NH_2_, -Cl, and -NO_2_) and phosphotungstic acids (PTA) (PTA@MIL-101-X) also showed enhanced catalyst-cellulose interactions (Figure 3). NO_2_-grafted catalyst PTA@MIL-101-NO_2_ achieved the highest glucose yield due to its highest affinity to cellulosic materials (Han et al., 2019). The efficiency of cellulose hydrolysis increases with the increase of the electronegativity (-NO_2_ > -Cl > -NH_2_ > -Br) of the binding groups (Han et al., 2019), which is consistent with the observation by Shuai and Pan (Shuai et al., 2012). A series of solid acid catalysts bearing -Cl were synthesized with different methods and enhanced glucose yields by about 2.5 times (93%) compared to the catalysts without -Cl (37%) (Pang et al., 2014; Shen et al., 2014; Zhao et al., 2014; Zuo et al., 2014; Hu et al., 2016; Yang et al., 2016; Shen et al., 2017; Shen et al., 2018; Ding et al., 2019; Li et al., 2020; Li et al., 2020). These catalysts follow the same binding mechanism that proposed above for promoting cellulose hydrolysis efficiency.

**FIGURE 4:**
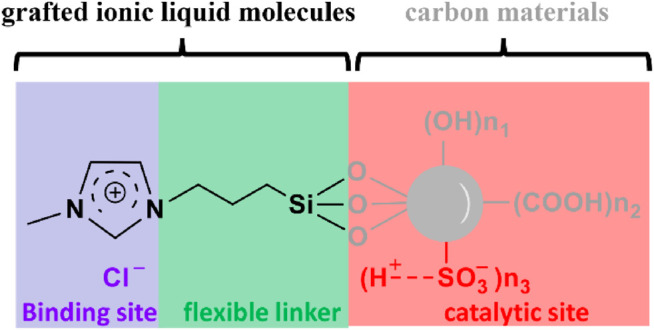
A sulfonated biochar material grafted with ionic liquid molecules as a cellulase-mimetic solid acid catalyst.

Ionic liquids consisting of electronegative anions such as -Cl and -Br that can effectively disrupt cellulose crystalline *via* forming new hydrogen bonds with cellulose hydroxyls are also potential candidates for cellulose-binding sites. A sulfonated biochar material grafted with ionic liquid molecules (e.g. 1-(trimethoxy propyl silane)-3-methylimidazolium chloride) could convert about 35.8% of microwave-pretreated bamboo(750 W) at 120°C in 2 h (Figure 4; Zhang et al., 2013). The IL groups flexibly joined to biochar, similar to the CBD and interdomain linker of cellulase, can efficiently break the hydrogen bonding network of cellulose crystalline and form new hydrogen bonds with cellulose hydroxyls (Figure 4). Therefore, ionic liquid molecules, compared to other binding sites discussed previously, can act as very promising binding sites for converting cellulose and real lignocellulosic biomass that contain crystalline structures. Such a unique property of ionic liquids is worth more attention in future work towards developing a more practical catalyst for biorefineries.

### (B) Binding *Via* Hydrophobic Interaction

**FIGURE 5:**
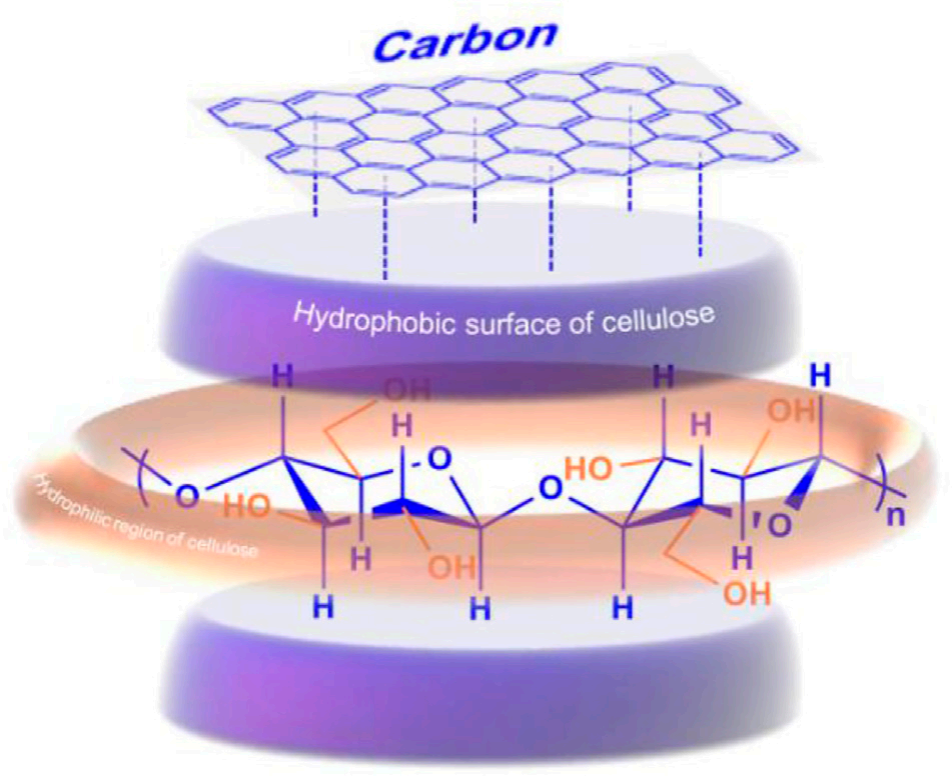
Adsorption of cellulose onto carbon materials *via* the hydrophobic interactions between the axial faces of cellulosic materials and the polycyclic aromatic rings of carbon materials.

Inspired by the hydrophobic interaction mechanism between cellulose and the CBD of cellulase, Mosier et al. screened amino acids that could bind cellulose. Indoles and tryptophan blue that have aromatic moieties were found to have high affinities to cellulose (Mosier et al., 2004), which highly indicates that hydrophobic interactions are essential for cellulose-cellulase binding other than hydrogen bonding interactions. Consistently, a variety of carbon materials demonstrate high adsorption capacity for cellulose *via* hydrophobic interactions (Chung et al., 2012; Chung et al., 2015; Onda et al., 2008; Yabushita et al., 2016). For example, a mesoporous carbon material with a pore diameter of 3.2 nm was able to adsorb soluble sugars (glucose and cello-oligosaccharides) in a quantity up to 670 mg g^−1^ (Chung et al., 2012); 1 g of a zeolite-templated carbon material (ZTC) could adsorb 800 mg of cellulosic molecules (Chung et al., 2015). The adsorption is mainly attributed to the CH–π hydrophobic interactions between the CH groups on the axial face of cellulose and the polycyclic aromatics of carbon materials, whereas no hydrogen bonding interaction between the mesoporous carbon materials and cellulosic materials was observed (Figure 5; Chung et al., 2012). The adsorption strength increased with the increasing number of glucose units (Chung et al., 2015), which is consistent with the study reported by Shuai and Pan (Shuai et al., 2012). Negative enthalpy change was observed during the adsorption of cello-oligosaccharides on carbon materials due to the CH–π interactions (Yabushita et al., 2014). The hydrophobic interaction between cellulose and carbon aromatic rings exists considerably only in the presence of water as a solvent because water molecules can drive the interaction of hydrophobic interfaces of cellulose and carbon materials (Meyer et al., 2006). The GO sheets in the carbon-based solid acids can also guide the assembly of cellulose *via* both π-π and hydrogen bonding interactions, facilitating the spread of glucose and reducing the occurrence of side reactions (Zhang et al., 2017).

### (C) Binding *Via* Reversible Chemical Bonding

**FIGURE 6:**
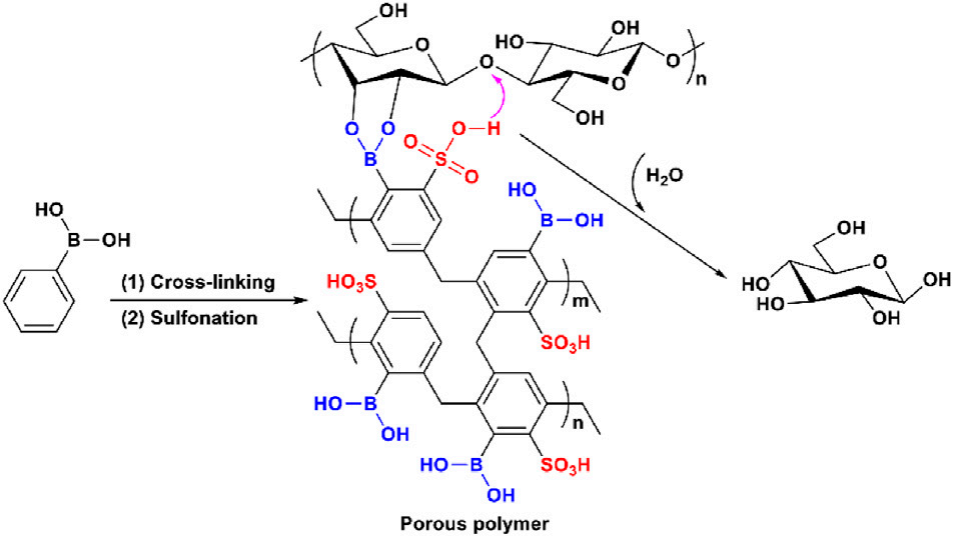
Proposed cellulose hydrolysis mechanism for the cellulase-mimetic catalyst bearing boronic and sulfonic acids on porous polymer.

Other than the aforementioned physical interactions, a reversible chemical binding strategy was also explored by Yang and Pan (Figure 6; Yang et al., 2016). Boronic acid can form reversible covalent bonds with the vicinal hydroxyls of carbohydrates (Springsteen et al., 2002; John Griffin et al., 2004; Matsumoto et al., 2005) and therefore can be used as the binding site of cellulase-mimetic solid acids. A bifunctional porous polymer bearing boronic and sulfonic acids achieved glucose yields of 43% in 24 h and 95% in 48 h, respectively, for cellulose hydrolysis (Figure 5). Specifically, the boronic acid group reacts with two vicinal hydroxyl groups of cellulose to form a reversible five-element ring structure, which brings cellulose close to the catalytic sites of the catalyst for hydrolysis.

## Perspectives

Cellulase-mimetic solid acid catalysts are excellent candidates to replace traditional solid acid catalysts, in which the catalytic sites and binding sites play a crucial role in efficient cellulose hydrolysis. Cellulase-mimetic solid acids usually demonstrate higher hydrolysis efficiencies than conventional solid acids without tailored catalytic and binding sites; however, most of the current studies use cellobiose, amorphous cellulose, or ball-milled cellulose as the substrate for catalyst performance testing. The selective hydrolysis of more practical substrates such as untreated cellulose that has crystalline structures and lignocellulosic biomass that has unremoved lignin is still challenging and problematic for such a type of solid catalysts due to the limited interaction between the solid catalysts and bulk cellulosic materials. A few attempts to use pretreated lignocelluloses as substrates only got limited glucose yields (almost all less than 20%, Table 1) (Zhang et al., 2012; Hu et al., 2014; Hu et al., 2016). To overcome the limitation of current catalysts for hydrolyzing crystalline cellulose and lignocellulosic materials, future research could be focused on green solvents or activators that can be developed as a cellulase-mimetic morphogenesis for disrupting the crystalline structure of cellulose prior to the binding (Brandt et al., 2013; da Costa Lopes et al., 2013; Lee et al., 2014).

Developing water-soluble cellulase-mimetic solid acid catalysts could be a potential solution to the slow mass transfer problem. The water solubility of such catalysts could facilitate the fast adsorption of water-soluble catalyst molecules to cellulose surface. This is particularly necessary for hydrolyzing cellulosic materials that exist in the solid form and with lignin. In this way, the water-soluble catalyst can be easily separated from lignin. With molecular design, temperature-, pH- and light-sensitive functional groups can be incorporated into the water-soluble catalysts to manipulate their solubility and absorptivity for the recovery of the catalysts after hydrolysis. The effects of lignin and hemicellulose on stability of cellulase-mimetic acid catalysts remains to be further studied.

The structure of the polyaromatic ring as a binding site of cellulase-mimetic catalysts has a great application prospect in future work. Although carbon materials have promising cellulose adsorption potential, the unique structural features of carbon polyaromatics are not fully optimized. Typically, the activity is defined by aromatic structure (Basal plane) and the specific functional groups on edges while other important properties of carbon such as electron density, hybridizations, defects, and electrophilicity are not well understood. The current advancement in material development may provide the internal local surface constrains for better interaction between the catalyst framework and cellulose in an intracrystalline free space for enhanced enzyme-like activity. Furthermore, a deeper understanding of the nature of the active and reactive sites could provide a pathway towards next-generation functionalized polyaromatic as a biomimetic catalyst for cellulose hydrolysis.

## References

[B1] Asensio J. L. Ardá A. Cañada F. J. Jiménez-Barbero J. (2012). Carbohydrate-aromatic Interactions. Acc. Chem. Res. 46, 946–954. 10.1021/ar300024d 22704792

[B2] Badieyan S. Bevan D. R. Zhang C. (2012). Probing the Active Site Chemistry of β-Glucosidases Along the Hydrolysis Reaction Pathway. Biochemistry 51, 8907–8918. 10.1021/bi300675x 23043218

[B3] Berlin A. Maximenko V. Gilkes N. Saddler J. (2007). Optimization of Enzyme Complexes for Lignocellulose Hydrolysis. Biotechnol. Bioeng. 97, 287–296. 10.1002/bit.21238 17058283

[B4] Brandt A. Gräsvik J. Hallett J. P. Welton T. (2013). Deconstruction of Lignocellulosic Biomass with Ionic Liquids. Green. Chem. 15, 550–583. 10.1039/C2GC36364J

[B5] Brown R. Jurasek L. (1979). Hydrolysis of Cellulose: Mechanisms of Enzymatic and Acid Catalysis. Carbohydr. Res. 181. 10.1021/ba-1979-0181

[B6] Chen Z. Li Q. Xiao Y. Zhang C. Fu Z. Liu Y. (2019). Acid-base Synergistic Catalysis of Biochar Sulfonic Acid Bearing Polyamide for Microwave-Assisted Hydrolysis of Cellulose in Water. Cellulose 26, 751–762. 10.1007/s10570-018-2098-3

[B7] Cho E. J. Lee S. J. Lee K. Lee D.-S. Lee Y. J. Bae H.-J. (2015). A Reusable Biomimetic Magnetic Nanoenzyme for Cellulosic Biomass Degradation. Bioenerg. Res. 8, 788–795. 10.1007/s12155-014-9559-9

[B8] Cho E. J. Song Y. Lee Y. J. Bae H.-J. (2016). Preparation and Characterization of Novel Green Magnetic Nanocatalyst for Cellulosic Biomass Degradation Under Mild Conditions. J. Ind. Eng. Chem. 40, 185–190. 10.1016/j.jiec.2016.06.022

[B9] Chung P.-W. Charmot A. Gazit O. M. Katz A. (2012). Glucan Adsorption on Mesoporous Carbon Nanoparticles: Effect of Chain Length and Internal Surface. Langmuir 28, 15222–15232. 10.1021/la3030364 23020524

[B10] Chung P.-W. Yabushita M. To A. T. Bae Y. Jankolovits J. Kobayashi H. (2015). Long-chain Glucan Adsorption and Depolymerization in Zeolite-Templated Carbon Catalysts. ACS Catal. 5, 6422–6425. 10.1021/acscatal.5b01172

[B11] da Costa Lopes A. M. João K. G. Morais A. R. C. Bogel-Łukasik E. Bogel-Łukasik R. (2013). Ionic Liquids as a Tool for Lignocellulosic Biomass Fractionation. Sustain. Chem. Process 1, 1–31. 10.1186/2043-7129-1-3

[B12] Ding X. Guo Y. Liu S. Gong Z. Dai X. Li N. (2019). High-efficiency Depolymerization of Microcrystalline Cellulose in 1-Butyl-3-Methylimidazolium Chloride Over a Magnetically Recoverable Cellulase-Mimetic Resin Catalyst. j biobased mat bioenergy 13, 389–394. 10.1166/jbmb.2019.1862

[B13] dos Santos A. C. Ximenes E. Kim Y. Ladisch M. R. (2019). Lignin-Enzyme Interactions in the Hydrolysis of Lignocellulosic Biomass. Trends Biotechnol. 37, 518–531. 10.1016/j.tibtech.2018.10.010 30477739

[B14] Gan L. Zhu J. Lv L. (2017). Cellulose Hydrolysis Catalyzed by Highly Acidic Lignin-Derived Carbonaceous Catalyst Synthesized via Hydrothermal Carbonization. Cellulose 24, 5327–5339. 10.1007/s10570-017-1515-3

[B15] Georgelis N. Yennawar N. H. Cosgrove D. J. (2012). Structural Basis for Entropy-Driven Cellulose Binding by a Type-A Cellulose-Binding Module (CBM) and Bacterial Expansin. Proc. Natl. Acad. Sci. 109, 14830–14835. 10.1073/pnas.1213200109 22927418 PMC3443152

[B16] Han J. Wang Y. Wan J. Ma Y. (2019). Catalytic Hydrolysis of Cellulose by Phosphotungstic Acid-Supported Functionalized Metal-Organic Frameworks with Different Electronegative Groups. Environ. Sci. Pollut. Res. 26, 15345–15353. 10.1007/s11356-019-04923-7 30929176

[B17] Hara M. (2010). Biomass Conversion by a Solid Acid Catalyst. Energy Environ. Sci. 3, 601–607. 10.1039/B922917E

[B18] Hu L. Li Z. Wu Z. Lin L. Zhou S. (2016). Catalytic Hydrolysis of Microcrystalline and rice Straw-Derived Cellulose Over a Chlorine-Doped Magnetic Carbonaceous Solid Acid. Ind. Crops Prod. 84, 408–417. 10.1016/j.indcrop.2016.02.039

[B19] Hu L. Lin L. Wu Z. Zhou S. Liu S. (2015). Chemocatalytic Hydrolysis of Cellulose into Glucose Over Solid Acid Catalysts. Appl. Catal. B: Environ. 174-175, 225–243. 10.1016/j.apcatb.2015.03.003

[B20] Hu S. Jiang F. Hsieh Y.-L. (2015). 1D Lignin-Based Solid Acid Catalysts for Cellulose Hydrolysis to Glucose and Nanocellulose. ACS Sust. Chem. Eng. 3, 2566–2574. 10.1021/acssuschemeng.5b00780

[B21] Hu S. Smith T. J. Lou W. Zong M. (2014). Efficient Hydrolysis of Cellulose Over a Novel Sucralose-Derived Solid Acid with Cellulose-Binding and Catalytic Sites. J. Agric. Food Chem. 62, 1905–1911. 10.1021/jf405712b 24512554

[B22] Huang A. A. (1975). Enzymatic Hydrolysis of Cellulose to Sugar. Biotechnol. Bioeng. Symp. 5, 245–252. 10.1007/978-1-4615-9290-7-84 1238128

[B23] Huang Y.-B. Fu Y. (2013). Hydrolysis of Cellulose to Glucose by Solid Acid Catalysts. Green. Chem. 15, 1095–1111. 10.1039/c3gc40136g

[B24] John Griffin G. Shu L. (2004). Solvent Extraction and Purification of Sugars from Hemicellulose Hydrolysates Using Boronic Acid Carriers. J. Chem. Technol. Biotechnol. 79, 505–511. 10.1002/jctb.1013

[B25] Kitano M. Yamaguchi D. Suganuma S. Nakajima K. Kato H. Hayashi S. (2009). Adsorption-Enhanced Hydrolysis of β-1,4-Glucan on Graphene-Based Amorphous Carbon Bearing SO3H, COOH, and OH Groups. Langmuir 25, 5068–5075. 10.1021/la8040506 19397353

[B26] Lee H. V. Hamid S. B. A. Zain S. K. (2014). Conversion of Lignocellulosic Biomass to Nanocellulose: Structure and Chemical Process. Scientific World J. 2014, 1–20. 10.1155/2014/631013 PMC4163452 25247208

[B27] Li H.-X. Zhang X. Wang Q. Yang D. Cao Q. Jin L. e. (2020). Study on the Hydrolysis of Cellulose with the Regenerable and Recyclable Multifunctional Solid Acid as a Catalyst and its Catalytic Hydrolytic Kinetics. Cellulose 27, 285–300. 10.1007/s10570-019-02777-3

[B28] Li H. X. Shi W. J. Zhang X. Liu P. Cao Q. Jin L. e. (2020). Catalytic Hydrolysis of Cellulose to Total Reducing Sugars with Superior Recyclable Magnetic Multifunctional MCMB‐based Solid Acid as a Catalyst. J. Chem. Technol. Biotechnol. 95, 770–780. 10.1002/jctb.6262

[B29] Matsumoto M. Ueba K. Kondo K. (2005). Separation of Sugar by Solvent Extraction with Phenylboronic Acid and Trioctylmethylammonium Chloride. Separat. Purif. Techn. 43, 269–274. 10.1016/j.seppur.2004.11.010

[B30] Meyer E. E. Rosenberg K. J. Israelachvili J. (2006). Recent Progress in Understanding Hydrophobic Interactions. Proc. Natl. Acad. Sci. 103, 15739–15746. 10.1073/pnas.0606422103 17023540 PMC1635073

[B31] Mission E. G. Quitain A. T. Sasaki M. Kida T. (2017). Synergizing Graphene Oxide with Microwave Irradiation for Efficient Cellulose Depolymerization into Glucose. Green. Chem. 19, 3831–3843. 10.1039/C7GC01691C

[B32] Mosier N. S. Wilker J. J. Ladisch M. R. (2004). Rapid Chromatography for Evaluating Adsorption Characteristics of Cellulase Binding Domain Mimetics. Biotechnol. Bioeng. 86, 756–764. 10.1002/bit.20104 15162451

[B33] Onda A. Ochi T. Yanagisawa K. (2008). Selective Hydrolysis of Cellulose into Glucose Over Solid Acid Catalysts. Green. Chem. 10, 1033–1037. 10.1039/B808471H

[B34] Orozco A. M. Al-Muhtaseb A. a. H. Albadarin A. B. Rooney D. Walker G. M. Ahmad M. N. M. (2011). Acid-catalyzed Hydrolysis of Cellulose and Cellulosic Waste Using a Microwave Reactor System. RSC Adv. 1, 839–846. 10.1039/C1RA00329A

[B35] Palkovits R. Tajvidi K. Procelewska J. Rinaldi R. Ruppert A. (2010). Hydrogenolysis of Cellulose Combining Mineral Acids and Hydrogenation Catalysts. Green. Chem. 12, 972–978. 10.1039/C000075B

[B36] Pang J. Wang A. Zheng M. Zhang T. (2010). Hydrolysis of Cellulose into Glucose Over Carbons Sulfonated at Elevated Temperatures. Chem. Commun. 46, 6935–6947. 10.1039/c0cc02014a 20730212

[B37] Pang Q. Wang L. Yang H. Jia L. Pan X. Qiu C. (2014). Cellulose-derived Carbon Bearing -Cl and -SO3H Groups as a Highly Selective Catalyst for the Hydrolysis of Cellulose to Glucose. RSC Adv. 4, 41212–41218. 10.1039/C4RA05520A

[B38] Qiao Y. Teng N. Zhai C. Na H. Zhu J. (2018). High Efficient Hydrolysis of Cellulose into Sugar by Chemical Catalytic Method. Prog. Chem. 30, 1415–1423. 10.7536/PC180126

[B39] Rabinovich M. L. Melnick M. S. Bolobova A. V. (2002). The Structure and Mechanism of Action of Cellulolytic Enzymes. Biochemistry 67, 850–871. 10.1023/a:1019958419032 12223085

[B40] Rinaldi R. Schüth F. (2010). Acid Hydrolysis of Cellulose as the Entry point into Biorefinery Schemes. ChemSusChem 3, 296. 10.1002/cssc.201090010 19950346

[B41] Shen F. Guo T. Bai C. Qiu M. Qi X. (2018). Hydrolysis of Cellulose with One-Pot Synthesized Sulfonated Carbonaceous Solid Acid. Fuel Process. Techn. 169, 244–247. 10.1016/j.fuproc.2017.10.015

[B42] Shen F. Smith R. L. Li L. Yan L. Qi X. (2017). Eco-friendly Method for Efficient Conversion of Cellulose into Levulinic Acid in Pure Water with Cellulase-Mimetic Solid Acid Catalyst. ACS Sust. Chem. Eng. 5, 2421–2427. 10.1021/acssuschemeng.6b02765

[B43] Shen S. Cai B. Wang C. Li H. Dai G. Qin H. (2014). Preparation of a Novel Carbon-Based Solid Acid from Cocarbonized Starch and Polyvinyl Chloride for Cellulose Hydrolysis. Appl. Catal. A: Gen. 473, 70–74. 10.1016/j.apcata.2013.12.037

[B44] Shimizu K.-i. Satsuma A. (2011). Toward a Rational Control of Solid Acid Catalysis for Green Synthesis and Biomass Conversion. Energ. Environ. Sci. 4, 3140–3153. 10.1039/C1EE01458G

[B45] Shuai L. Pan X. (2012). Hydrolysis of Cellulose by Cellulase-Mimetic Solid Catalyst. Energ. Environ. Sci. 5, 6889–6894. 10.1039/c2ee03373a

[B46] Springsteen G. Wang B. (2002). A Detailed Examination of Boronic Acid-Diol Complexation. Tetrahedron 58, 5291–5300. 10.1016/S0040-4020(02)00489-1

[B47] Sugano Y. Vestergaard M. d. C. Saito M. Tamiya E. (2011). A Carbon Nanotube Structured Biomimetic Catalyst for Polysaccharide Degradation. Chem. Commun. 47, 7176–7188. 10.1039/c1cc10927h 21607239

[B48] Suganuma S. Nakajima K. Kitano M. Yamaguchi D. Kato H. Hayashi S. (2008). Hydrolysis of Cellulose by Amorphous Carbon Bearing SO3H, COOH, and OH Groups. J. Am. Chem. Soc. 130, 12787–12793. 10.1021/ja803983h 18759399

[B49] Toda M. Takagaki A. Okamura M. Kondo J. N. Hayashi S. Domen K. (2005). Biodiesel Made with Sugar Catalyst. Nature 438, 178. 10.1038/438178a 16281026

[B50] Van de Vyver S. Peng L. Geboers J. Schepers H. de Clippel F. Gommes C. J. (2010). Sulfonated Silica/carbon Nanocomposites as Novel Catalysts for Hydrolysis of Cellulose to Glucose. Green. Chem. 12, 1560–1563. 10.1039/c0gc00235f

[B51] Verma D. Tiwari R. Sinha A. K. (2013). Depolymerization of Cellulosic Feedstocks Using Magnetically Separable Functionalized Graphene Oxide. RSC Adv. 3, 13265–13272. 10.1039/C3RA41025K

[B52] Wang J. Xi J. Wang Y. (2015). Recent Advances in the Catalytic Production of Glucose from Lignocellulosic Biomass. Green. Chem. 17, 737–751. 10.1039/c4gc02034k

[B53] Wang S. Sima G. Cui Y. Chang L. Gan L. (2020). Efficient Hydrolysis of Cellulose to Glucose Catalyzed by Lignin-Derived Mesoporous Carbon Solid Acid in Water. Chin. J. Chem. Eng. 28, 1866–1874. 10.1016/j.cjche.2020.03.012

[B54] Yabushita M. Kobayashi H. Fukuoka A. (2014). Catalytic Transformation of Cellulose into Platform Chemicals. Appl. Catal. B: Environ. 145, 1–9. 10.1016/j.apcatb.2013.01.052

[B55] Yabushita M. Kobayashi H. Hasegawa J.-y. Hara K. Fukuoka A. (2014). Entropically Favored Adsorption of Cellulosic Molecules onto Carbon Materials through Hydrophobic Functionalities. ChemSusChem 7, 1443–1450. 10.1002/cssc.201301296 24644071

[B56] Yabushita M. (2016). “Mechanistic Study of Cellulose Hydrolysis by Carbon Catalysts,” in A Study on Catalytic Conversion of Non-food Biomass Intochemicals: Fusion of Chemical Sciences and Engineering. Editor Yabushita M. (Singapore: Springer Singapore), 77–112. 10.1007/978-981-10-0332-5_3

[B57] Yabushita M. Techikawara K. Kobayashi H. Fukuoka A. Katz A. (2016). Zeolite-templated Carbon Catalysts for Adsorption and Hydrolysis of Cellulose-Derived Long-Chain Glucans: Effect of Post-synthetic Surface Functionalization. ACS Sust. Chem. Eng. 4, 6844–6851. 10.1021/acssuschemeng.6b01796

[B58] Yang Q. Pan X. (2016). Bifunctional Porous Polymers Bearing Boronic and Sulfonic Acids for Hydrolysis of Cellulose. ACS Sust. Chem. Eng. 4, 4824–4830. 10.1021/acssuschemeng.6b01102

[B59] Yang Q. Pan X. (2021). Introducing Hydroxyl Groups as Cellulose-Binding Sites into Polymeric Solid Acids to Improve Their Catalytic Performance in Hydrolyzing Cellulose. Carbohydr. Polym. 261, 117895. 10.1016/j.carbpol.2021.117895 33766380

[B60] Yang Q. Pan X. (2016). Synthesis and Application of Bifunctional Porous Polymers Bearing Chloride and Sulfonic Acid as Cellulase-Mimetic Solid Acids for Cellulose Hydrolysis. Bioenerg. Res. 9, 578–586. 10.1007/s12155-015-9702-2

[B61] Yu F. Smet M. Dehaen W. Sels B. F. (2016). Water-soluble Sulfonated Hyperbranched Poly(arylene Oxindole) Catalysts as Functional Biomimics of Cellulases. Chem. Commun. 52, 2756–2759. 10.1039/C5CC08742B 26759837

[B62] Zechel D. L. Withers S. G. (2000). Glycosidase Mechanisms: Anatomy of a Finely Tuned Catalyst. Acc. Chem. Res. 33, 11–18. 10.1021/ar970172+ 10639071

[B63] Zhang C. Fu Z. Dai B. Zen S. Liu Y. Xu Q. (2013). Chlorocuprate Ionic Liquid Functionalized Biochar Sulfonic Acid as an Efficiently Biomimetic Catalyst for Direct Hydrolysis of Bamboo Under Microwave Irradiation. Ind. Eng. Chem. Res. 52, 11537–11543. 10.1021/ie401100x

[B64] Zhang C. Fu Z. Liu Y. C. Dai B. Zou Y. Gong X. (2012). Ionic Liquid-Functionalized Biochar Sulfonic Acid as a Biomimetic Catalyst for Hydrolysis of Cellulose and Bamboo Under Microwave Irradiation. Green. Chem. 14, 1928–1935. 10.1039/c2gc35071h

[B65] Zhang C. Wang H. Liu F. Wang L. He H. (2013). Magnetic Core-Shell Fe3O4@C-So3h Nanoparticle Catalyst for Hydrolysis of Cellulose. Cellulose 20, 127–134. 10.1007/s10570-012-9839-5

[B66] Zhang M. Wu M. Liu Q. Wang X. Lv T. Jia L. (2017). Graphene Oxide Mediated Cellulose-Derived Carbon as a Highly Selective Catalyst for the Hydrolysis of Cellulose to Glucose. Appl. Catal. A: Gen. 543, 218–224. 10.1016/j.apcata.2017.06.033

[B67] Zhao X. Wang J. Chen C. Huang Y. Wang A. Zhang T. (2014). Graphene Oxide for Cellulose Hydrolysis: How it Works as a Highly Active Catalyst. Chem. Commun. 50, 3439–3442. 10.1039/c3cc49634a 24535471

[B68] Zhou Z. Liu D. Zhao X. (2021). Conversion of Lignocellulose to Biofuels and Chemicals via Sugar Platform: An Updated Review on Chemistry and Mechanisms of Acid Hydrolysis of Lignocellulose. Renew. Sust. Energ. Rev. 146, 111169. 10.1016/j.rser.2021.111169

[B69] Zhu J. Gan L. Li B. Yang X. (2016). Synthesis and Characteristics of Lignin-Derived Solid Acid Catalysts for Microcrystalline Cellulose Hydrolysis. Korean J. Chem. Eng. 34, 110–117. 10.1007/s11814-016-0220-5

[B70] Zhu S. Xu J. Cheng Z. Kuang Y. Wu Q. Wang B. (2020). Catalytic Transformation of Cellulose into Short Rod-like Cellulose Nanofibers and Platform Chemicals Over Lignin-Based Solid Acid. Appl. Catal. B: Environ. 268, 118732–118744. 10.1016/j.apcatb.2020.118732

[B71] Zuo Y. Zhang Y. Fu Y. (2014). Catalytic Conversion of Cellulose into Levulinic Acid by a Sulfonated Chloromethyl Polystyrene Solid Acid Catalyst. ChemCatChem 6, 753–757. 10.1002/cctc.201300956

